# Non-health outcomes affecting self-care behaviors and medical decision-making preference in patients with type 2 diabetes: a cross-sectional study

**DOI:** 10.1186/s12911-020-1095-2

**Published:** 2020-04-23

**Authors:** Ming-Jye Wang, Hung-Ming Lin, Li-Chen Hung, Yi-Ting Lo

**Affiliations:** 1 Department of Secretariat, National Taiwan University Hospital Hsin-Chu Branch, No.25, Lane 442, Sec.1, Jingguo Rd, Hsinchu City, 300 Taiwan; 2 Department of Healthcare Management, Yuanpei University of Medical Technology, Hsinchu, Taiwan; 3 Department of Business Administration, Minghsin University of Science and Technology, Hsinchu, Taiwan; 4 Department of Public Health, China Medical University, Taichung, Taiwan; 5 Department of Development and Planning, National Taiwan University Hospital Hsin-Chu Branch, Hsinchu, Taiwan

**Keywords:** Health literacy, Self-efficacy, Patient empowerment, Self-care behaviors, Type 2 diabetes

## Abstract

**Background:**

The effects of patient sustained self-care behaviors on glycemic control are even greater than the effects of medical treatment, indicating the value of identifying the factors that influence self-care behaviors. To date, these factors have not been placed in a single model to clarify the critical path affecting self-care behaviors. The aims of this study were to explore the relationships of these factors and the differences in patient preference for medical decision-making.

**Methods:**

A cross-sectional study was conducted among outpatients with type 2 diabetes at a regional teaching hospital. Purposive sampling was adopted to recruit 316 eligible patients via self-administered questionnaires. Partial least squares structural equation modeling was used for analysis.

**Results:**

Significant direct pathways were identified from health literacy to self-efficacy, patient empowerment, and self-care behaviors; from self-efficacy to self-care behaviors; and from patient empowerment to self-care behaviors. Indirect pathways were from health literacy to self-care behaviors via self-efficacy or patient empowerment. The pathway from health literacy to self-efficacy was significantly stronger in those preferring shared decision-making than in those who preferred physician decision-making.

**Conclusions:**

Health literacy is a critical factor in improving self-care behaviors in patients with type 2 diabetes, and the effect of health literacy on self-efficacy was more significant in the shared decision-making than in the physician decision-making. Therefore, developing an effective health strategy to strengthen health literacy awareness and designing friendly, diverse health literacy materials, and application tools is the most important factor to facilitate self-care behaviors in this population.

## Background

Diabetes is a life-long chronic condition that leads to serious consequences. Over 425 million people worldwide are currently living with diabetes [1], with 1.6 million deaths directly attributed to diabetes each year [2]. Therefore, the World Health Organization (WHO) calls on countries to prevent or delay the onset of diabetes by promoting a healthy lifestyle, to reduce the risk of diabetes and effectively manage the disease to decrease the rates of complications and mortality [3].

To effectively control the progression of diabetes, prevent long-term complications, and promote quality of life, patients with diabetes need effective self-care behaviors (SCB) [4], which have an even greater impact than medical treatment [5]. SCB refers to “decisions and actions that an individual can take to cope with a health problem or to improve his or her health” [6]; according to the American Association of Diabetes Educators (AADE) [7] defined the AADE 7 Self-Care Behaviors™ that included healthy eating, being active, monitoring, taking medication, problem solving, healthy coping, and reducing risks. Effective self-care behaviors are necessary to maintaining optimal HbA1C levels [8], particularly in patients with type 2 diabetes (T2DM) [9, 10]. In recent years, there has been a call for health care evaluations to move beyond the measurement of health outcomes, and to consider the value of non-health outcomes, such as empowerment, psychosocial outcomes, and quality of life [11]. Therefore, clarifying the impact of non-health outcomes on SCB is an urgent issue.

Health literacy (HL) is defined as “the degree to which individuals have the capacity to obtain, process, and understand basic health information and services needed to make appropriate health decisions” [12]. It is increasingly recognized as an important modifiable psychosocial factor in the self-management behaviors of patients with T2DM [13], and is also theorized to be an important non-clinical factor in decreasing the risk of adverse outcomes in diabetes [14]. HL is considered to be positively correlated with SCB [13, 15, 16], and can also affect SCB via self-efficacy [17, 18]. Therefore, HL is one of widely used in studying patient-related predictors of health behaviors [19].

Another factor well known to be associated with SCB in patients with diabetes is self-efficacy (SE), which is “the belief in one’s capacity to organize and execute the courses of action required to manage a prospective situation” [20]. SE is the main factor that directly affects health behaviors [21]. Patients with high SE have better compliance with SCB [22], both general SE and disease-specific SE can affect SCB [23]. To improve the SCB of patients with diabetes, high SE is necessary.

Diabetes self-management is a complex lifelong journey whose prerequisites for success are the patient’s active and responsible participation in the process. However, adopting and sustaining self-management practices may not always be easy. Then, motivation is an important factor in self-management, especially intrinsic motivation is more important than extrinsic motivation [24]. Intrinsic motivation refers to “engaging in a behavior or an activity for its own sake and personal rewards”. Extrinsic motivation refers to “performing a behavior or an activity to earn a reward or avoid punishment” [25]. Patient empowerment (PE) is a process designed to facilitate self-directed behavior change [26]. Therefore, PE is also one of another widely researched determinant of healthy behaviors [19]. The WHO defines empowerment as “a process through which people gain greater control over decisions and actions affecting their health” [27], which demonstrates that PE “doesn’t mean ‘giving’ people power. Rather it’s about ‘enabling’ them to recognize and use their power” [27]. Chen et al. [28] have found out that the empowerment approach has positive impact on improving SE and SCB. Patients with different health literacy levels may respond differently to PE. The Health Empowerment Model [29] proposes that PE is deeply interwoven with HL to affect the health outcomes of patients. Findings of the interactions between PE and HL in terms of health outcomes in patients are not consistent [30].

As mentioned above, patients with diabetes require a high level of responsibility and promise to implement a new lifestyle of SCB. HL, SE, and PE have been reported to directly or indirectly affect SCB [13, 15, 16, 23, 29], but they have not yet been integrated into a single model to identify their influence on SCB, including differences in patient preferences for medical decision-making. This study used partial least squares structural equation modeling (PLS-SEM) to analyze these relationships. The aims of the current study were to: 1) investigate the relationships between HL, SE, and PE on SCB in patients with T2DM; and 2) compare the differences in these relationships by preference in making medical decisions. These findings may provide a more effective reference to guide professional interventions, and improve SCB in patients with T2DM; thereby, helping these patients achieve effective blood glucose control and avoid complications associated with uncontrolled disease.

## Methods

### Study participants

The study was conducted with the participation of outpatients who were diagnosed with T2DM for more than 1 year (primary diagnosis included up to three diagnostic codes in the International Classification of Diseases, Ninth Revision, Clinical Modification: 250) upon visiting the Department of Metabolism of a regional teaching hospital in Hsinchu City, Taiwan. Purposive sampling was adopted to recruit eligible patients with consent during all clinic sessions from June through September 2017. Researchers described the purpose of this study briefly prior before acquiring written informed consent and distributing the questionnaires. A total of 372 questionnaires were distributed, and 316 completed self-administered questionnaires were collected, for a rate of valid questionnaires of 85%. The questionnaires include the scales of HL, SE, PE, and SCB, and the patient’s preference in making medical decisions.

### Research scale design

Data were derived from patients’ self-administered questionnaires. The questionnaire for this study was developed with reference to pre-existing validated scales and was adapted to conditions in this region, including the medical environment and terms understandable for patients. They were reviewed by an expert panel whose members included a specialist physician, dietitian, and health educator. The items included in the scale were selected for applicability and ease of administration. The details of each scale are as follows.

The HL scale was developed with reference to the Functional Communicative and Critical Health Literacy (FCCHL) scale developed by Ishikawa et al. [31]. The scale consists of 14 items in three dimensions: functional health literacy (FHL), 5 items; interactive health literacy (IHL), 5 items; and critical health literacy (CHL), 4 items. Each item was scored on a 5-point Likert scale (from 1 = strongly disagree to 5 = strongly agree). Mean scores of HL were obtained by summing the 14 item scores and dividing by the number of items, with higher scores indicating higher HL.

The SE scale used in this study was developed based on the Diabetes Self-Efficacy Scale [32] and Perceived Diabetes Self-Management Scale [33], which included 19 items on a 5-point Likert scale (from 1 = strongly disagree to 5 = strongly agree). Mean scores of SE were obtained by summing the 19 item scores and dividing by the number of items, with higher scores indicating higher SE.

The PE scale used in this study referred to the Chinese Diabetes Empowerment Process Scale [34], which included 15 items with 5-point Likert scale (from 1 = strongly disagree to 5 = strongly agree). Mean scores of PE were obtained by summing the 15 item scores and dividing by the number of items, with higher scores indicating higher PE.

The SCB scale used in this study was developed with reference to the Diabetes Self-Management Questionnaire [35], containing 14 items on 5-point Likert scale (from 1 = strongly disagree to 5 = strongly agree). Mean scores of SCB were obtained by summing the 14 item scores and dividing by the number of items, with higher scores indicating higher SCB.

The patient’s preference decision scale was based on the Patient’s Role Preference in Decision-Making questionnaire [36]. Patients selected their preference for making medical decisions, selecting one of these five items: 1 = like to make treatment decisions on their own; 2 = like to make treatment decisions on their own after listening to physician’s opinion; 3 = like to make treatment decisions together with the physician; 4 = like physician to make treatment decisions after talking to the patient; and 5 = like physician to make treatment decisions alone. Upon analysis, patients were categorized into three groups: patient decision-making (answers 1 and 2), shared decision making (SDM) (answer 3), and physician decision-making (answers 4 and 5).

### Data analysis

The PLS-SEM incorporates into canonical correlation concepts the important statistical analysis techniques of regression analysis, principal component analysis, and path analysis. PLS-SEM can be applied to Mediation and Moderation analysis, and has gained universal attention in the field of health care [37]. It can not only help the researcher handle measurement problems of variable reduction but also addresses the structural problems of predicting and interpreting relationships in the research hypothesis. In interpreting the relationship of the latent variable to the dependent variable in regression analysis, this type of analysis is not affected by multi-collinearity, being distribution-free, requires only a small sample size, and can be applied to either formative or reflective measurements [38]. PLS-SEM was used to investigate the relationships between HL, SE, and PE on SCB in patients with T2DM. The patient’s preferences in decision-making primarily included SDM and physical decision-making, in order to test the effects of moderation on differences in preference for medical decision-making, a multi-group analysis was conducted by comparing differences in coefficients of the corresponding structural paths for the constructs. A *p-*value of 0.05 or lower indicated a significant difference between groups. Descriptive statistics, including mean, standard deviations, and frequency, were used to analyze the distribution of patient characteristics, HL, SE, PE, SCB, and decision preferences. SmartPLS 3.0 (Institute of Operations Management and Organizations, University of Hamburg, Germany) was used for data analysis.

## Results

### Study participant characteristics

Among the 316 valid self-administered questionnaires, 39.6% were male and 60.4% were female. Those aged ≥65 years accounted for 63.3% of the study population; 44.7% had an elementary school education and 22.0% had a high school education. In terms of decision-making preferences, 56.3% preferred physician decision-making and 43.7% preferred SDM (Table 1).

**Table 1:** Participant’s characteristics

	N	%		N	%
Sex			Patient’s preference decision		
Male	125	39.6	SDM	138	43.7
Female	191	60.4	Physician decision	178	56.3
Age			Educational		
≦54	45	14.2	Elementary school	136	44.7
55–64	71	22.5	Middle school	54	17.8
65–74	103	32.6	High school	67	22.0
≧75	97	30.7	College or high	47	15.5

*N* number of participants; *SDM* shared decision making

### Reliability and validity

**Fig. 1:**
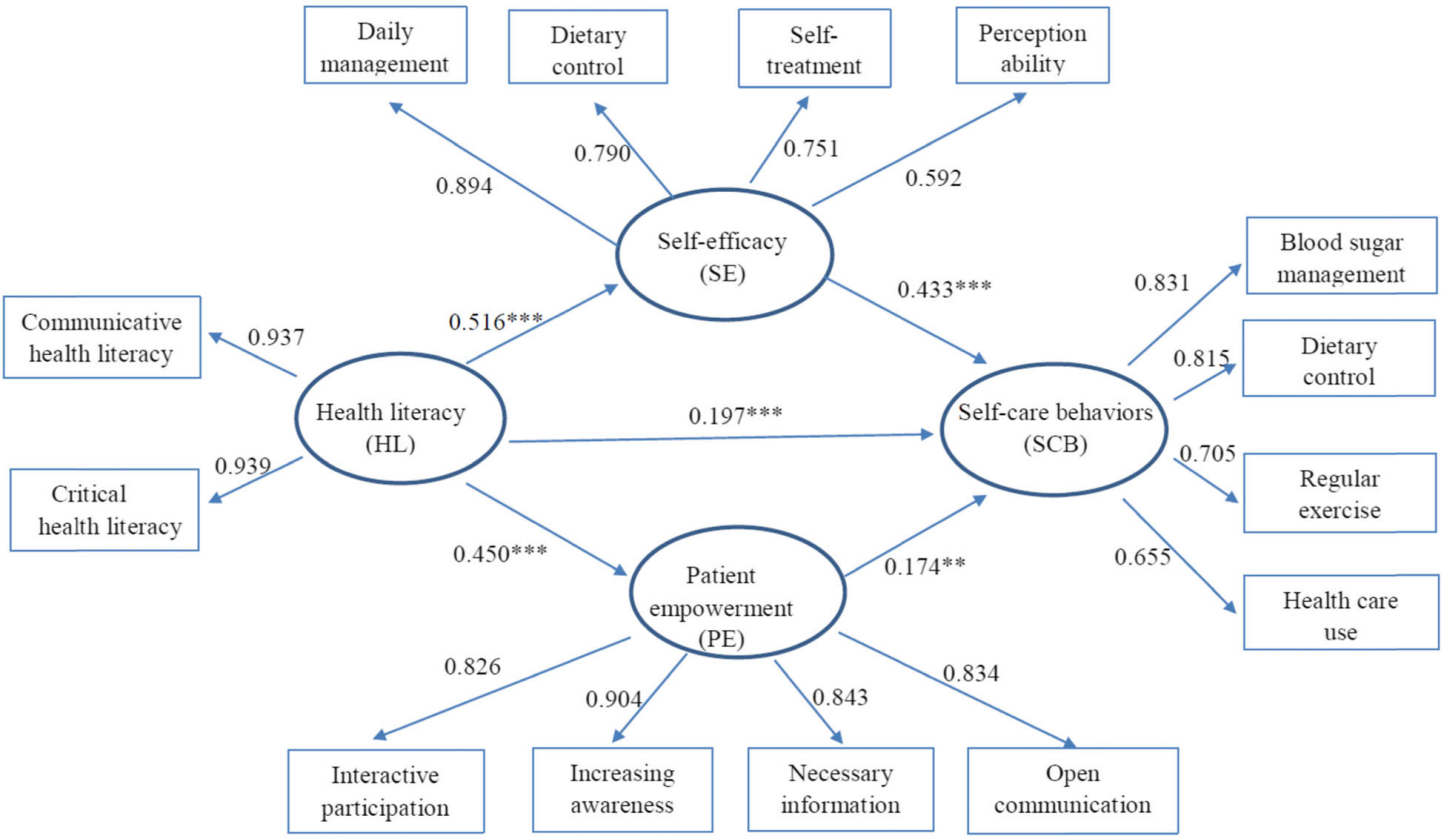
Path model of health literacy (HL), self-efficacy (SE), and patient empowerment (PE) on self-care behaviors (SCB). **P < 0.01; ***P < 0.001

In this study results were analyzed by measurement mode. Three criteria, including internal consistency, indicator reliability, and average variance extracted (AVE), were used to assess the convergent validity of each construct. The factor loadings of each dimension were between 0.592 and 0.939 (Fig. 1). The factor loading of FHL was 0.339. According to Hair et al. [39], the factor loading of each dimension is recommended to be > 0.5, so the FHL dimension was excluded from measurement mode analysis. Table 2 shows the Cronbach’s α and Composite Reliability (CR) values of each scale were greater than 0.7, which indicated good construct reliability and high internal consistency [40, 41]. The AVE was greater than 0.5, showing convergent validity [42]. Fornell and Larcker’s test shows that the correlation coefficient was lower than the value of the diagonal element ($\sqrt{\mathrm{AVE}}$), indicating that the measurement mode had the required discriminant validity [43].

**Table 2:** Reliability, convergent, and discriminant validity of measurement model

Construct	Mean	SD	correlation				Cronbach’s α	CR	AVE
			HL	SE	PE	SCB			
HL	3.42	0.47	0.938				0.863	0.936	0.880
SE	3.82	0.47	0.516	0.764			0.754	0.846	0.584
PE	4.03	0.48	0.450	0.638	0.853		0.876	0.914	0.727
SCB	3.66	0.52	0.499	0.646	0.539	0.755	0.762	0.840	0.570

*SD* standard deviation; *HL* health literacy; *SE* self-efficacy; *PE* patient empowerment; *SCB* self-care behaviors; *CR* composite reliability; *AVE* Average variance extracted

The scales were scored on 5-point Likert scale, with average values ranging from 3.42 to 4.03, indicating that participants had a more positive attitude towards each scale, especially PE (Table 2).

### Relationships between health literacy, self-efficacy, and patient empowerment on self-care behaviors

To clarify the path of HL, SE, and PE on SCB, this study applied the PLS Bootstrap method with 5000 resamplings to obtain inference statistics. As shown in Table 3, HL had a direct positive effect on SE (path coefficient 0.516, t value = 8.755, *p* < 0.001), PE (path coefficient 0.450, t value = 9.749, p < 0.001), and SCB (path coefficient 0.197, t value = 3.705, p < 0.001). SE had a direct positive effect on SCB (path coefficient 0.433, t value = 6.526, p < 0.001) and PE had a direct positive effect on SCB (path coefficient 0.174, t value = 2.890, *p* < 0.01). HL also indirectly influenced SCB via SE and PE (for SE: path coefficient 0.223, t value = 4.766, p < 0.001; for PE: path coefficient 0.079, t value = 2.890, p < 0.01). The relationships are shown in Fig. 1.

**Table 3:** Direct and indirect effects

Path	Path coefficient	t value	*P* value
Direct effect			
HL → SE	0.516	8.755	0.000^***^
HL → PE	0.450	9.749	0.000^***^
HL → SCB	0.197	3.705	0.000^***^
SE → SCB	0.433	6.526	0.000^***^
PE → SCB	0.174	2.890	0.004^**^
Indirect effect			
HL → SE → SCB	0.223	4.766	0.000^***^
HL → PE → SCB	0.079	2.890	0.004^**^

^**^p < 0.01; ^***^p < 0.001

### Comparing differences in relationships by preference in medical decision-making

To compare the relationships differences in terms of preference decisions (SDM and physician decision), Hensler et al. [44] pointed out that Measurement Invariance Assessment is necessary, which includes three steps: configural invariance, compositional invariance, and the equality of composite mean values and variances. Using the Measurement Invariance Assessment in SmartPLS usually automatically establishes configural invariance (Step 1). Table 4 shows the correlation coefficient of Step 2. In Step 3, the *p* value of both the mean value and the variance were greater than 0.05, which is not significant. These results show that the measurement invariance was established for the scales when measuring different groups.

**Table 4:** Results of the measurement invariance assessment

Construct	Step 2		Step 3			
	Correlation coefficient	P value	Variance	P value	Mean value	P value
HL	1.000	0.508	0.155	0.567	0.179	0.125
SE	0.996	0.141	0.383	0.088	−0.086	0.397
PE	0.998	0.447	0.029	0.883	−0.064	0.545
SCB	0.997	0.508	0.160	0.578	−0.069	0.520

Non-Significance at *p* value > 0.05 show the measurement invariance was established

In order to test the effects of moderation on preference in medical decision-making differences (SDM and physician decision), a partial least square multi-group analysis was conducted by comparing differences in coefficients of the corresponding structural paths for the constructs. *P* values of 0.05 or lower indicated that there were significant differences between the paths in the groups [45]. The results demonstrated that of the five paths, HL positively affecting SE was significantly stronger for the SDM group than for the physician decision group (Table 5, path coefficient 0.249, *p* < 0.01).

**Table 5:** Multi-Group Analysis

Path	Path coefficient difference (SDM Group-physician decision Group)	*P* value
HL → SE	0.249	0.008^**^
HL → PE	0.144	0.061
HL → SCB	0.171	0.919
SE → SCB	0.012	0.453
PE → SCB	0.060	0.322

*SDM* shared decision making. ^**^Significance at *p* value < 0.01

Results for a one-sided test

## Discussion

This study is the first to place HL, SE, PE, and SCB in patients with T2DM in the same model to clarify their relationships using PLS-SEM, as well as to test the effect of HL on SE was more significant in the SDM group than in the physician decision-making group.

These findings clarify the relationships of HL, SE, and PE to SCB, which are: HL directly positively influences SE and SCB, and SE directly positively influences SCB. HL can also indirectly influence SCB through SE. These relationships were consistent with previous researches [8, 17, 18]. Patients with higher HL can better promote their own health-related behaviors, and they may feel more confident in their ability to complete SCB [46, 47]. In particular, IHL and CHL have a greater impact on SE than FHL [8]. The more patients can enhance their SE, the more they may feel empowered to handle their situation [48], so HL plays an important role in the impact of SCB, and SE is also an important predictor of SCB [23].

This study also further clarified the effect of HL and PE on SCB, which is: HL directly positively influences PE, and PE directly positively influences SCB. HL can also indirectly influence SCB through PE. Studies have shown that HL and PE are deeply interwoven [22], and each independently affects SCB [49], but limited HL is a threat to PE and self-management [50]. Wang et al. [30] proposed that PE may promote SCB in patients with high IHL and CHL, but may have no effect on SCB in patients with low communicative and critical health literacy (CCHL). Obviously, no matter patients are empowered externally or internally, these empowerment may sustained only when patients have adequate HL. Increasing HL is an antecedent of PE [51–53]. Strengthening PE without adequate HL may lead patients to harm their health condition by making uninformed decisions, and HL plays a bigger role than PE in determining health status [19].

In this study, the mean PE was 4.03 and the mean SE was 3.82, indicating that patients tended to have high PE and SE. The combined care plans for patients with T2DM under the National Health Insurance System in Taiwan encourages patients to be empowered by health providers. Participating physicians, health educators, and dieticians must be certified to implement this plan in order to assist patients undergoing regular medical treatment and self-health management, and follow the patients’ medical regimen. The components of the initial or continuing care visit include a medical history, physical examination, laboratory evaluation, management plan, and diabetes self-management education, so PE and SE are generally high. However, the mean of the HL scale was 3.42, indicating that patients’ HL was obviously insufficient, and the mean of the SCB scale (3.66) was not high as well. Although these patients had the beliefs and actions to perform health behaviors and wanted to control their own health behaviors, they still felt a strong sense of powerlessness. Therefore, the self-management behaviors of the patients relied too heavily on the health care system to take active responsibility for SCB. The mindset of these patients must be changed, and their self-improvement in HL is the cornerstone by which to promote SCB.

In terms of the differences in preference in decision-making (SDM vs physician decision), HL directly positively affected SE in the five paths of Fig. 1 significantly more for the SDM group than for the physician decision group. Because HL can improve the ability of the patient to perform SCB, the patient then is better able to participate in SDM [54], further influencing clinical decisions. Patients will be more confident to take on self-management when they have more health-related knowledge, feel they can seek out resources and applications, and have positive interactions with health care professionals. As a result, the self-efficacy of patients is also improved. The study by Brabers et al. [54] showed that HL was associated with patient involvement in SDM, especially CHL. Patients participating in SDM have an increased commitment to health behaviors [55] and greater awareness and confidence to start their treatment [56]. Also, because patients with HL are more likely to play an active role in clinical decision-making, patients with HL are much more likely to show behavioral change [57]. Because HL involves obtaining, processing, and understanding health information for all aspects of health care, such as prevention, screening, diagnosis, and treatment, it is considered the basis of the health care delivery system [58].

Limitations of this study include the fact that participant inclusion was based on patient consent, which may have introduced selection bias into the study sample. This study selected only a single regional hospital, which may be a limitation of extrapolation the data. Also, the sample included only outpatients with T2DM; those with other types of diabetes, severity, duration of diabetes, and morbidity or more advanced disease, may have different outcomes. SCB was evaluated using a questionnaire and was not measured objectively. Further study is needed to explore the specific factors that influence HL in order to improve the level of HL.

## Conclusions

This study provides insight into HL as the most important factor in the facilitation of self-care behaviors in patients with T2DM, and clarified the relationships between HL, SE, and PE on SCB. HL was found to have a direct positive effect on SE, PE, and SCB. HL also had an indirectly positive influence in SCB through SE and PE. Separately, SE and PE each directly positively affected SCB. In the SDM group, HL directly positively affected SE significantly more strongly than in the physician decision group. Therefore, developing an effective health strategy to strengthen health literacy awareness and designing friendly, diverse health literacy materials and application tools is necessary. In addition, promoting SDM to improve health outcomes and reduce complications in patient with T2DM is also necessary.

## Data Availability

The data was collected from the patient’s self-administered questionnaire with patient consent during the current study. The datasets are not publicly available, but are accessible from the corresponding author on reasonable request.

## Abbreviations

WHO: World Health Organization
SCB: Self-care behaviors
HbA1C: Glycated Hemoglobin
T2DM: Type 2 diabetes
HL: Health literacy
SE: Self-efficacy
PE: Patient empowerment
PLS-SEM: Partial least squares structural equation modeling
FCCHL: Functional Communicative and Critical Health Literacy
FHL: Functional health literacy
IHL: Interactive health literacy
CHL: Critical health literacy
SDM: Shared decision making
CR: Composite Reliability
AVE: Average Variance Extracted
CCHL: Communicative and critical health literacy
